# Interference-free Determination of Carbamazepine in Human Serum Using High Performance Liquid Chromatography: A Comprehensive Research with Three-way Calibration Methods

**Published:** 2017

**Authors:** Shiva Ghafghazi, Taraneh Moini Zanjani, Maryam Vosough, Masoumeh Sabetkasaei

**Affiliations:** a *Department of Pharmacology, Neuroscience Research center, Faculty of Medicine, Shahid Beheshti University of Medical Sciences, Evin, Tehran, Iran.*; b *Chemistry and Chemical Engineering Research Center of Iran, P.O. Box 14335-186 Tehran, Iran.*

**Keywords:** Carbamazepine, High-performance liquid chromatography, Multi-way algorithms, Serum

## Abstract

In the present study, a comprehensive and systematic strategy was described to evaluate the performance of several three-way calibration methods on a bio-analytical problem. Parallel factor analysis (PARAFAC), alternating trilinear decomposition (ATLD), self-weighted alternating trilinear decomposition (SWATLD), alternating penalty trilinear decomposition (APTLD), and unfolded partial least squares combined with the residual bilinearization procedure (U-PLS/RBL) were applied on high performance liquid chromatography with photodiode-array detection (HPLC-DAD) data to quantify carbamazepine (CBZ) in different serum samples. Using the proposed approach, successfully quantification of CBZ in human plasma, even in the presence of diverse uncalibrated serious interfering components was achieved. Moreover, the accuracy and precision of each algorithm for analyzing CBZ in serum samples were compared using root mean square error of prediction (RMSEP), the recovery values and figures of merits and reproducibility of the analysis. Satisfying recovery values for the analyte of interest were obtained by HPLC-DAD on a Bonus-RP column using an isocratic mode of elution with acetonitrile/K2HPO4 (pH = 7.5) buffer solution (45:55) coupled with second-order calibrations. Decreas of the analysis time and less solvent consumption are some of the pluses of this method. The analysis of real samples showed that the modeling of complex chromatographic profiles containing CBZ as the target drug using any of the mentioned algorithms can be potentially benefit drug monitoring in therapeutic research.

## Introduction

Carbamazepine (5-H-dibenzo (b,f) azepine-5-carboximide) is an antiepileptic drug which is used for treatment of epilepsy and mental disorders (1). This drug has numerous effects on the environment and human. Hence, evaluating the side effects of this medicine is very important and vital (2-3). Despite of the reported properties of this medicine, there are some analgesic effects concerning carbamazepine prescription (4). Due to different therapeutic index of carbamazepine in human serum, measuring low concentrations of carbamazepine will be useful for clinical purposes and pharmacokinetic studies (5). 

Qualitative and quantitative analysis of trace amounts of pharmaceuticals in biological samples, with sufficient accuracy and precision has been a challenging task for analytical purposes, and several analytical methods have been proposed for detecting and quantifying pharmaceuticals in biologic samples. In optimal condition, the extraction processes are not selective, therefore; the sample interferences must be removed. In addition, there are various fundamental challenges with chromatographic data such as the presence of noise, background effect, the contribution of the displacement of the retention times and also peak overlaps, which have significant effects on qualitative and quantitative results (6). Isolation of confounding species is a costly and time consuming process. In addition, green chemistry and less use of organic solvents were important to lead the study (7-8). Multivariate resolution methods have been presented in recent decades for the solution of the basic problems that occur in the analysis of complex mixtures in chromatography (9-13). These methods have the second-advantage property, which brings about to determine the analyte in the presence of interfering species (14). Most of the chromatographic data doesn’t follow the trilinear structure. In addition, deviation from linearity may result in changes in the retention time and the shape of the chromatographic peaks. Consequently, correction of the retention times shifts for the former problem and using a standard addition methodology for the second problem are necessary before applying the trilinear methods (14). Generally, there are two ways to deal with non-trilinearity: the first way is modeling the data arrays with PARAFAC2 (15), U-PLS/RBL (16) and multivariate curve resolution–alternating least squares (MCR-ALS) (17) methods, so there is no need to correct the displacement in retention time. Applying alignment algorithms as a pre-processing step for the chromatographic peaks and taking the advantages of the trilinear algorithms, such as PARAFAC, ATLD, SWATLD, APTLD is the second way to resolve the mentioned problem. Among the various trilinear decomposition methods, PARAFAC has been frequently taken into consideration, this close attention results from the unique response of this method (18-21) Indeed, PARAFAC emphasizes on fitting the basic data and acceptable results will be observed only when the true number of species is estimated (22-23). On the other hand, if there is higher estimation, aberrant and invalid results will be obtained (24-26). ATLD, SWATLD, and APTLD methods can model the data arrays with a fast convergence speed and the advantage of being insensitive to component number. On the other hand, it was found that baseline elimination is a fundamental pre-treatment step to decrease the data complexity and detect unknown species in the samples (27). Totally, it has been shown that retention time shift correction together with baseline subtraction steps improves the performance of second order algorithms (28-31). 

The final algorithm is based on the combination of residual bilinearization and bilinear least square (BLLS/RBL) (32-33) or U-PLS/PBL (34). Arguably, MCR/ALS method (35-36) which has the second-order advantage is a repetitive method to resolve the three-dimensional data. In this research topic, few papers have compared second-order calibration algorithms (37-40) or have reviewed these algorithms (41-42). In a recent study, the authors used MCR/ALS method for simultaneous determination of carbamazepine and phenobarbital in human serum samples in a fast way (43). In fact, acceptable analytical results through MCR/ALS modeling necessitate providing good initial estimates of profiles. On the other hand, one should be aware of probable range of feasible solution because of rotational ambiguity (disadvantage of MCR/ALS) and evaluate the retrieved profiles for confirming their uniqueness (36).

Hence, in this study, a comprehensive study was performed for quantitative measuring of carbamazepine in human serum samples with the emphasis on comparing the performance of three-way techniques such as PARAFAC, ATLD, SWATLD, APTLD, and U-PLS/RBL. In order to evaluate the real specimens, serum samples of 21 morphine addicted patients who had received carbamazepine before the surgery were collected and analyzed with mentioned methods. 

## Experimental

*Materials*

Carbamazepine (purity higher than 98%) was obtained from pharmaceutical company Daroopakhsh (Tehran, Iran). HPLC-grade methanol (MeOH), acetonitrile (ACN) and ethyl acetate (EA) were from Merck (Germany). Human serum was obtained from Taleghani Medical Centre (Tehran, Iran) and stored at −20 ^◦^C in the refrigerator. Morphine-dependent serum samples which were received from faculty of medicine, Shahid Beheshti University of medical sciences, were taken from 21 patients with three groups of patients who had received carbamazepine before surgery. The samples were collected before surgery, 2 h and 12 h after surgery, respectively. Phosphoric acid, di-potassium hydrogen phosphate, and sodium hydroxide were of analytical grade from Merck. Nylon membranes filters with the pore size of 0.22 µM (Varian, USA) were used for filtering solvents, calibration and real samples before HPLC analysis.Stock solution of CBZ (200 µg mL^-1^) was prepared by accurately weighting the required amounts of the compound, and dissolving in methanol and storing at -4 °C in dark glass vials. This solution was stable for at least a month. Standard working mixtures, at different concentrations, were prepared daily by appropriate dilution of the stock solution with mobile phase. 

*Equipment*

A 320R centrifuge with cooling system (Hettich, Germany) and a Sonorex Digital 10 P ultrasonic bath (Germany) were used. HPLC was performed using an Agilent 1200 Series system equipped (Agilent Technologies Inc., USA), composed of a Rheodyne 7725 manual injector, a degasser, a quaternary pump, a column oven compartment, and a Hewlett-Packard 1200 series photo diode-array detector (DAD). Agilent Chemstation software (version B.03.01) was used for data processing and acquisition. The column oven temperature was set at 25 ºC and chromatographic separation was carried out on a Bonus-RP column (15 cm × 0.46 cm, 5 µM particle size, Agilent). 

*Chromatographic conditions*

The chromatography was performed by applying an isocratic mobile mode, consisting acetonitrile (45%, v/v) and 0.005 mol L^-1^ K_2_HPO_4_ (pH = 7.5) buffer solution (55%, v/v). The mobile phase flow rate was 1.0 mL min^-1 ^and the injection volume was 20 μL. DAD detector was set to record between 210-400 nm with the spectral resolution of 1.5 nm and integration period of 0.4 s per spectrum. Total run time was less than 5 min. 

Data which was collected by Chemstation software (B.03.01), exported as Microsoft Excel^®^ file for further processing. All calculations were done using MATLAB (version 7.2.0.232 R2006a, The Mathworks, Natick, MA), using the MVC2 routine, a structured MATLAB toolbox for second-order calibration developed by Olivieri (44).

*Sample handling and extraction procedure*

Five hundred microliters of serum samples were mixed with 500 µL of 0.1 M NaOH and 3 mL of ethyl acetate. The mixture was vortexed for 30 s and then centrifuged at 4000 rpm for 10 min. The supernant was transferred into a clean tube and 1 mL of ethyl acetate was added to the sample and was centrifuged again for 5 min. The combined organic layers were evaporated to dryness under a stream of nitrogen. Finally, the residue was dissolved in 500 µL of mobile phase in ultrasonic bath and filtered through a 0.22 µM PTFE syringe filter and an aliquot of 20 µL etwas injected into the HPLC system. 

*Calibration and prediction samples*

The concentration levels for calibration samples of CBZ correspond to values in rang of 0.1-9.1 µg mL^-1^, according to the recent publication (43). A set of five samples of CBZ was prepared in pure solvent and was used as the calibration set. Then, a set of thirteen blank serum samples from two different pools were spiked with suitable amounts of standard CBZ and treated as explained in previous section. The concentration levels of the drug (0-8.15 µg mL^-1^) were selected considering the levels usually found in serum of different patients for therapeutic drug monitoring purposes. 

## Results and discussion

*General considerations*

**Figure 1:**
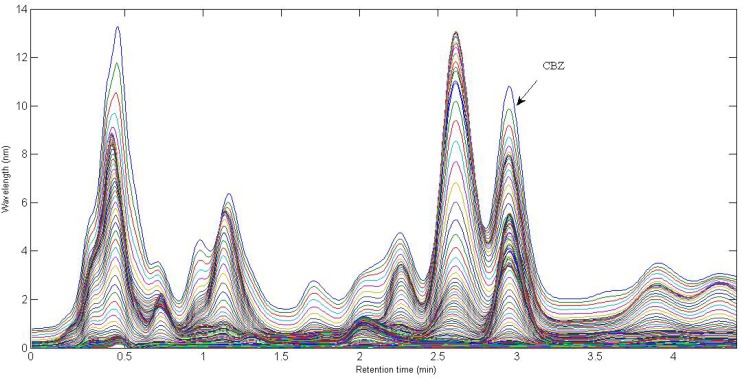
Chromatographic profiles, each at a single wavelength (225-370 nm), for a typical spiked serum sample with 2.35 µg L^-1^ of CBZ. The analyte of interest are indicated

**Figure 2:**
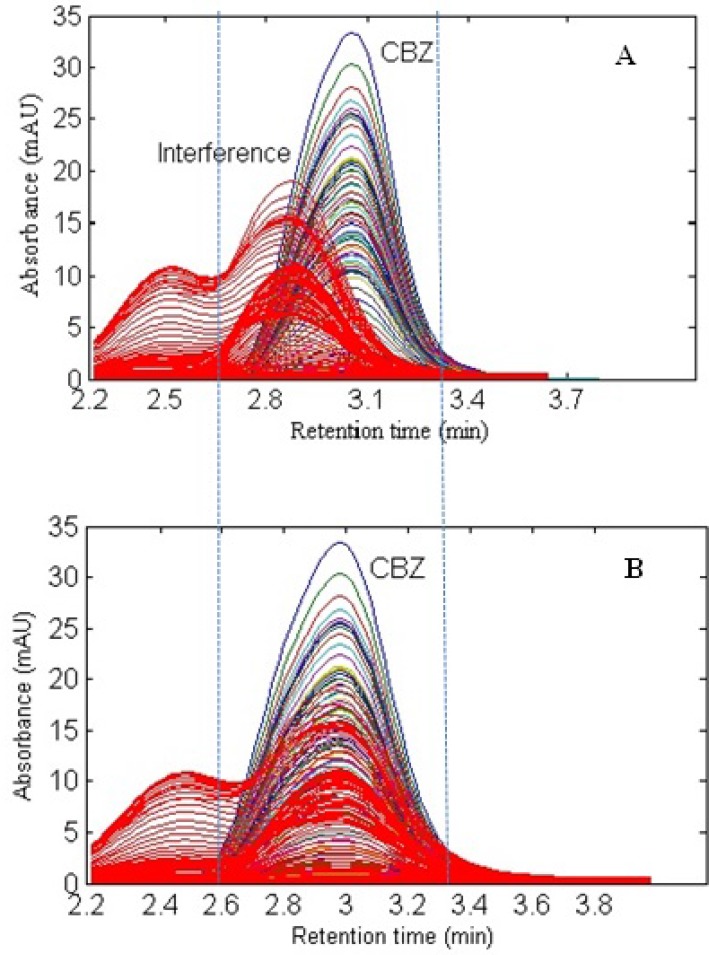
Overlay chromatograms including CBZ and interference (A) before elution time shifts correction and (B) after alignment

In the present work, a simple mobile phase of acetonitrile-0.005 mol L^-1^ K_2_HPO_4_ (pH = 7.5) buffer solution (45:55, v/v) was selected (45). With this mobile phase composition, the retention time of CBZ was 2.97 min. Figure 1. shows the chromatographic profiles recorded at multiple wavelengths for one of the spiked serum samples (S5). As can be seen, in addition to a baseline offset, there is apparently coelution problem in this sample, which confirms the appearance of interfering components from the biological sample. So, the studied information space is not analyte specific. In these cases, the analyte of interest can be quantified through univariate calibration by changing the experimental conditions, optimization of mobile phase condition to longer run times. However, these involve spending time and resources and strongly depend on the sample matrix. One of the most important points in this respect is the fact that changing the experimental or instrumental will not generate any guarantee to ensure that the separation of the future samples will be complete. Second-order calibration methods which model three-way data are today useful alternatives to deal with this challenging chromatographic data by exploiting second-order advantage. For the present problem at hand, a suitable subset of each data matrix in each way of measurement was selected. This contains the chromatographic region of 2.59-3.15 min and the spectral region of 225-370 nm. The constructed three-way data arrays was supposed to be analyzed with the aid of the trilinear algorithms of PARAFAC, ATLD, SWATLD and APTLD and also the latent variable based algorithm of U-PLS/RBL. On the other hand, due to the retention time shift between the calibration and serum samples and consequence invalid results, some preprocessing such as chromatographic alignment of all data matrices with a reference matrix is necessary before applying trilinear algorithms. Several retention time shift correction methods have been proposed in the literature (46-48). In the present study, multivariate rank alignment method proposed by Prazen (46) was applied. An example of retention time shift correction can be appreciated from Figures 2(A) and (B). 

In addition, the necessary of retention time shift correction, the presence of background drift which was different for different serum samples needed the attention. In the current study, the complexity of the real sample data was reduced as a separate step by background elimination for two-dimensional signals based on asymmetric least squares splines regression approach (49). Furthermore, determination of appropriate number of responsive components is essential. In this study, the optimum number of the components was selected based on SVD and core consistency diagnostic (CORCONDIA) test (25). 

**Table 1:** Predicted concentrations of CBZ using multiway algorithms on two different serum samples spiked with different amount of analytes

Sample	CBZ concentrations (μg mL^−1^)^a^					
Serum 1	Taken	ATLD	SWATLD	APTLD	PARAFAC	U-PLS/RBL
Unspiked	-	n.d^b^	n.d	n.d	n.d	n.d
s1 s2 s3 s4 s5	0.48 0.95 7.60 5.45 2.35	0.39(80.4%) 0.74(78.4%) 6.78(89.3%) 5.03(93.3%) 2.23(95.1%) [6.71]^c^	0.63(133.0%) 0.95(99.7%) 6.23 (82.0%) 4.88(89.5%) 2.17(92.4%) [5.24]	0.63(133.0%) 0.95(99.7%) 6.23 (82.0%) 4.88(89.5%) 2.17(92.4%) [5.24]	0.37(78.0%) 0.75(79.2%) 6.91 (91.0%) 5.02(92.2%) 2.34(99.7%) [5.60]	0.38(80.4%) 0.61 (64.0%) 6.04(79.4%) 3.78 (69.3%) 1.72 (73.0%) [6.22]
Serum 2						
Unspiked	-	n.d.	n.d.	n.d.	n.d.	n.d.
s6 s7 s8 s9 s10 s11 s12	0.85 8.15 4.12 6.45 3.20 0.50 1.80	0.92(108.2%) 6.52 (80.0%) 4.93(119.6%) 5.33 (82.6%) 2.99 (93.4%) 0.47 (94.0%) 1.79 (99.8%) [12.53]^c^	0.92(108.2%) 6.79 (83.3%) 4.33(105.1%) 5.35 (82.9%) 3.03 (94.7%) 0.50 (100.0%) 1.79 (99.80%) [9.23]	0.92(108.2%) 6.79 (83.3%) 4.33(105.1%) 5.34 (82.8%) 3.03 (94.7%) 0.41 (82.0%) 1.79 (99.8%) [6.68]	0.93(109.4%) 7.37 (90.4%) 3.07 (74.5%) 5.65 (87.6%) 3.11 (97.2%) 0.62 (124.0%) 1.79 (99.8%) [2.82]	0.96(113.3%) 7.23(88.7%) 3.51(85.2%) 5.51(85.4%) 3.06(95.6%) 0.53(105.3%) 1.69(93.6%) [6.85]

a Recoveries in parenthesis.

b Not detected.

c RSD (%) for three replicates of s5 and s12 in square brackets.

**Table 2:** Figures of merit and statistical validation results for the determination of CBZ in serum by ATLD, SWATLD, APTLD, PARAFAC and U-PLS/RBL

**Parameter **	**ATLD**	**SWATLD**	**APTLD**	**PARAFAC**	**U-PLS/RBL**
**REC±SD**	92.84±12	97.56 ±14	95.99 ±14	93.59±13	86.10±14
**RMSEP**	1.597	0.670	0.672	0.507	0.913
**LODs (µg ml** ^-1^ **)**	0.037	0.037	0.033	0.023	0.096
**LOQs (µg mL** ^-1^ **)**	0.124	0.124	0.111	0.076	0.320

**Table 3:** Results of quantification of CBZ on three groups of morphine-dependent patients’ serum samples by ATLD, SWATLD, APTLD, PARAFAC and U-PLS/RBL

**Group1** **samples** a	**ATLD**	**PARAFAC**	**SWATLD**	**APTLD**	**U-PLS/RBL**
Serum 1	0.70	0.90	0.80	0.80	0.70
Serum 2	0.08	0.05	0.082	0.083	0.17
Serum 3	0.08	0.08	0.09	0.09	0.15
Serum 4	0.11	0.06	0.16	0.16	0.12
Serum 5	0.09	0.09	0.11	0.11	0.07
Serum 6	0.16	0.14	0.19	0.19	0.17
Serum 7	0.11	0.07	0.14	0.14	0.10
Mean c	0.19±0.22	0.19±0.25	0.22±0.25	0.22±0.31	0.21±0.22
**Group2** **samples** a	**ATLD**	**PARAFAC**	**SWATLD**	**APTLD**	**U-PLS/RBL**
Serum 1	0.87	0.75	1.07	1.07	0.7
Serum 2	0.19	0.23	0.36	0.36	0.2
Serum 3	0.2	0.42	0.43	0.43	0.1
Serum 4	0.47	0.67	0.65	0.65	0.3
Serum 5	0.41	0.42	0.63	0.63	0.67
Serum 6	0.71	0.8	0.69	0.88	0.7
Serum 7	0.70 [3.05]^b^	0.75[2.75]	0.95[2.46]	0.95[2.46]	0.53[5.13]
Mean c	0.51±0.26	0.58±0.25	0.68±0.22	0.71±0.26	0.46±0.20
**Group3** **samples** a	**ATLD**	**PARAFAC**	**SWATLD**	**APTLD**	U-PLS/RBL
Serum 1	0.81	0.83	1.05	1.05	0.7
Serum 2	0.2	0.23	0.39	0.39	0.2
Serum 3	0.21	0.23	0.43	0.43	0.20
Serum 4	0.39	0.41	0.68	0.68	0.40
Serum 5	0.34	0.36	0.54	0.54	0.20
Serum 6	0.57	0.23	0.75	0.75	0.50
Serum 7	0.96	0.96	1.01	1.01	0.80

a Groups 1-3 samples, correspond to serum samples of morphine-dependent patients, before surgery, 2 h and 12 h after surgery, respectively.

b RSD (% ) for three replicates analysis of sample 7 (group 2) in square bracket.

c Mean concentration values and standard deviation obtained for determination of CBZ in each group of patients.

**Figure 3:**
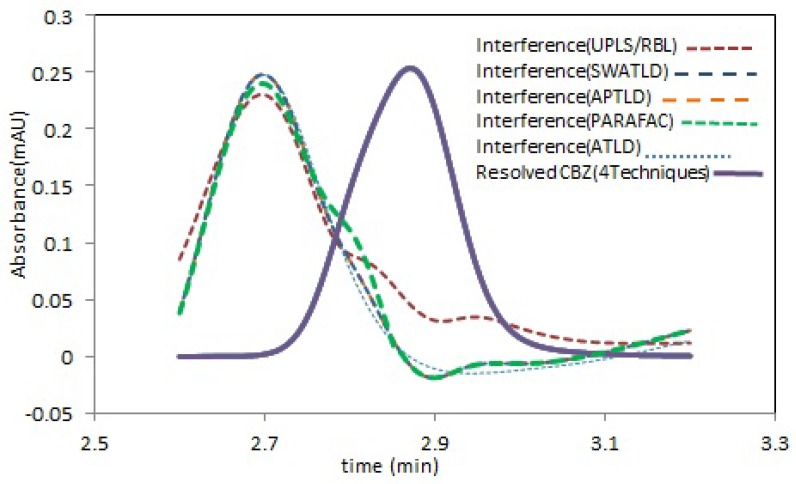
Estimated elution time profiles retrieved by all techniques analysis this region containing CBZ (purple solid line) and interfering compound. (Color figure available online

**Figure 4:**
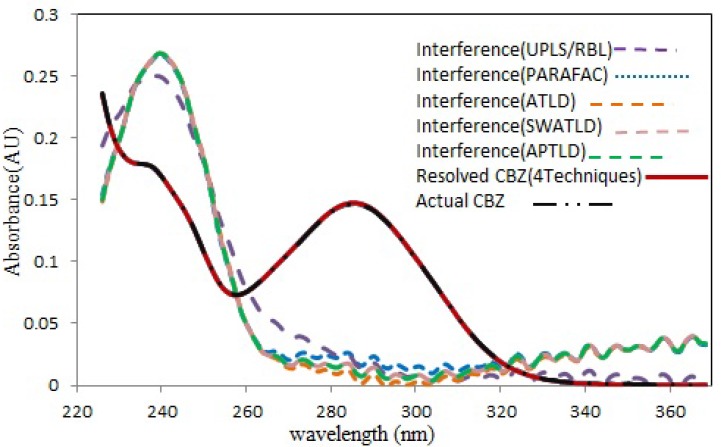
Spectral profiles recovered by all techniques modeling for CBZ. Comparison between the normalized pure analyte spectra for CBZ (black dot line) and the spectra reconstructed by the all techniques (red solid line). The interfering components have been shown for CBZ. (Color figure available online

**Figure 5:**
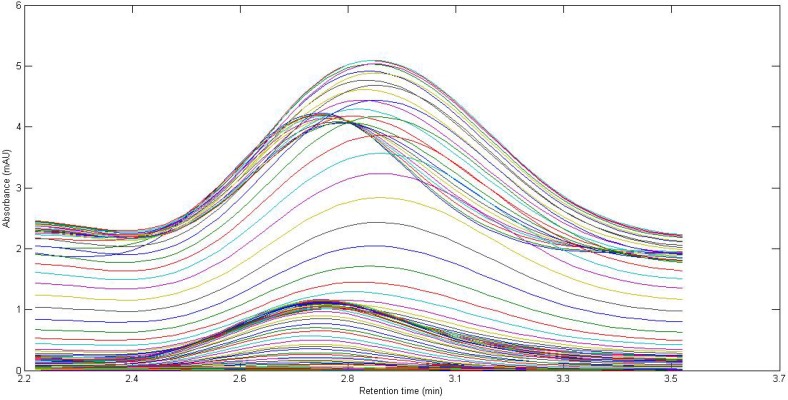
Second-order HPLC-DAD data of a patient’s serum sample.

**Figure 6.**
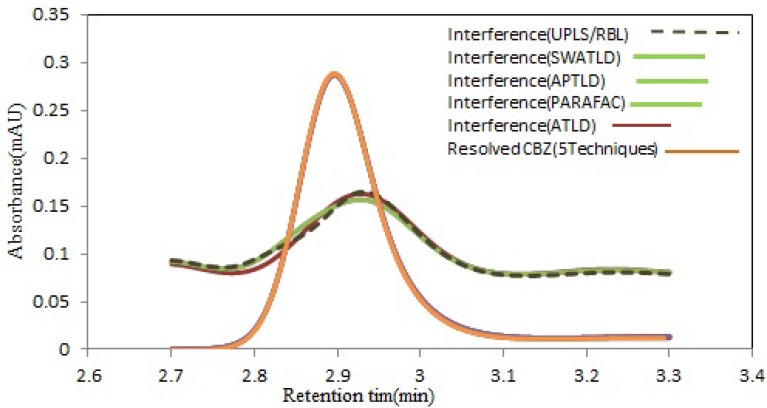
Estimated chromatographic profiles by all techniques modeling for morphine-dependent serum sample (serum1) which includes CBZ (orang solid line) and one interfering component. (Color figure available online

*Validation samples modelling with second-order calibration algorithms*

Figure 1. shows the chromatogram of a typical serum sample spiked with CBZ in multiple wavelengths (225-370 nm). It can be observed that at least one interfering component is eluted before CBZ. The chromatographic pattern confirmed the fact that classical univariate calibration is not a suitable and reliable method of quantification in this condition. As a matter of fact, different complexity problems were faced in this study during the analysis of different serum samples, which was completely evident and justified the application of second-order multivariate calibration methods. 

Three-way data arrays were constructed from multiple HPLC-DAD matrices including 15 calibration matrices and each of validation samples. Hence, the size of each cube of data submitted to multiway analysis was (16×91)×96. So, the resolution and quantification information belong to each sample was obtained each time from decomposition process. Quantification performed after completing the decomposition process for every unknown sample. Then, after confirming that component model belongs to the analyte, the concentration value of the target analyte *f* was obtained by regression of the first *I* elements of **a**_I__+1,f_ against the standard concentration values of** y**_f_ through a pseudo-univariate calibration curve: 

$\beta_{y_{f}}+[a_{1,f}|\cdots |a_{1,f}]$

[1]

where β_f_ is the slope of the least squares fitting and "+" shows the pseudoinverse. The estimated concentration in the unknown sample a_I__+1_th is:

$Y_{u,f}=\frac{a_{I}+1,f}{\beta_{f}}$

[2]

The predicted concentrations results, with the mentioned algorithms for CBZ, have been shown in Figure 3 and good agreement between the predicted values and the real spiked concentrations is clear.

Figure 4 shows the resolved spectral profiles by the mentioned algorithms. As can be seen, there is a perfect correlation between the recovered and the normalized pure spectrum of CBZ. Also, acceptable quantitative results were obtained (Table 1) for both spiked serum samples (serum 1 and 2), which is a further confirm for the effectiveness and accuracy of the mentioned techniques. For all cases the number of factors was 2 or 3, but never 1, which is normally required and presupposed for traditional univariate calibration. The mean recovery values through application of the mentioned algorithms for modeling 13 serum samples from two different pools were shown in Table 1. For all algorithms, the relative standard deviations (RSD%) of predicted concentration values for three replicates of s5 and s12 samples can be considered acceptable considering this fact that no attempt has been performed to remove the interfering compounds before HPLC analysis. 

Table 2. shows the statistical parameters such as root-mean-square-error of prediction (RMSE) and the figures of merit obtained through application of the algorithms for CBZ in serum samples using external calibration strategy. Both the limits of detection (LODs) and limits of quantification (LOQs) were obtained by all algorithms in the serum samples which were acceptable considering that a very simple methodology is being applied to a complex real system. Also, comparing RMSEP, RSD and LOD values obtained for validation samples showed that the PARAFAC provides slightly better results compared to aforementioned algorithms. Consequently, obtained recoveries by all algorithms were acceptable, so these algorithms can be eligible for some real applications, such as clinical analysis and pharmacokinetic investigations for patients. Also, taking the typical values found in serum samples into account, the proposed method can be directly applied without a pre-concentration step.

*Quantitative analysis of CBZ in real samples*

Since evaluation of the present method in analysing real samples is the most important purpose of the present study, a set of 21 serum samples belonging to three groups of morphine-dependent patients who have received carbamazepine before surgery, was analyzed using three way algorithms in three time intervals of before surgery, 2 h, and 12 h after surgery. 

Patient›s serum matrices contained different number of interfering compounds. As it can be observed in Figure 5, overlapping between the signals for this drug and serum components is clear. The analysis of CBZ was done by applying these algorithms to the sub-matrices containing CBZ peak. The results are shown in Table 3. As it is clear, there is an almost good agreement between the results obtained by these methods. Figure 6 shows the resolved chromatographic profiles through decomposition of HPLC-DAD data array using five mentioned algorithms for serum 1 (group 1) with the two factors. As can be appreciated from this figure, there is a perfect resemblance between the predicted profiles obtained via the algorithms. Also, the recovered chromatographic (Figure 6) and spectral profiles for unexpected components using U-PLS/RBL (figure has not been shown) was very similar to the serum samples. The test of repeatability was performed for one of the most complex samples with two interfering components. Therefore, the RSD% values for analysis of serum 7 (group 2) with three replicates was obtained as 3.05% for ATLD, 2.75% for PARAFAC, 2.46% for SWATLD and APTLD and 5.13% for U-PLS/RBL. This Table confirms that the concentration of CBZ increased before surgery until 2 h and remained nearly constant until 12 h after surgery.

## Conclusion

Different second-order calibration algorithms of PARAFAC, ATLD, SWATLD, APTLD and U-PLS/RBL were used in this paper, for modeling HPLC-DAD data sets. In comparing the multiway methodologies for resolving a complex bio-analytical problem, the following characteristics should be considered: analytical performance of the method, model interpretability as well as ease and speed of program operation. It has been shown that all methods are very helpful for direct determination of CBZ in complex matrices such as human serum despite the serious interferences. PARAFAC can provide the most stable result only if the selected component number is correct, but its convergence speed is slow. ATLD can obtain more information in extracting the trilinear structure in the data, while having the advantage of being insensitive to component number and fast convergence speed. SWATLD employing the same ideology as ATLD, inherits the advantage of insensitive to component number and fast speed. Larger penalty factors make APTLD close to SWATLD. U-PLS/RBL benefits from some important advantages. In fact, it is a rather straight forward technique having simpler and faster program operations. It is independent from initial estimate (guess) of the component spectra and shows good analytical performance. On the other hand, despite the other multiway methods, it is not possible to give a clear physical interpretation of the system using U-PLS/RBL modeling.

Finally, regarding ease of computer operation, all methods can be applied through a graphical user interfaced (GUI) in MATLAB environment. However, trilinear algorithms need extra pre-processing step of trilinearity correction in addition to background removal. So, U-PLS/RBL seems to be simpler considering the number, initial estimates requirements, and constraining parameters. 
